# Are Sex Differences in Preferences for Physical Attractiveness and Good Earning Capacity in Potential Mates Smaller in Countries With Greater Gender Equality?

**DOI:** 10.1177/1474704919852921

**Published:** 2019-05-30

**Authors:** Lingshan Zhang, Anthony J. Lee, Lisa M. DeBruine, Benedict C. Jones

**Affiliations:** 1 Institute of Neuroscience & Psychology, University of Glasgow, Glasgow, United Kingdom

**Keywords:** sex differences, mate preferences, gender inequality, attractiveness, status

## Abstract

On average, women show stronger preferences for mates with good earning capacity than men do, while men show stronger preferences for physically attractive mates than women do. Studies reporting that sex differences in mate preferences are smaller in countries with greater gender equality have been interpreted as evidence that these sex differences in mate preferences are caused by the different roles society imposes on men and women. Here, we attempted to replicate previously reported links between sex differences in mate preferences and country-level measures of gender inequality in a sample of 3,073 participants from 36 countries (data and code available at https://osf.io/4sr5f/). Although women preferred mates with good earning capacity more than men did and men preferred physically attractive mates more than women did, we found little evidence that these sex differences were smaller in countries with greater gender equality. Although one analysis suggested that the sex difference in preferences for good earning capacity was smaller in countries with greater gender equality, this effect was not significant when controlling for Galton’s problem or when correcting for multiple comparisons. Collectively, these results provide little support for the social roles account of sex differences in mate preferences.

Sex differences in human mate preferences have been widely reported in the literature on human mating strategies. That women tend to show stronger preferences for long-term mates with good earning capacity than men do, while men tend to show stronger preferences for physically attractive mates than women do, is a particularly robust finding (see Buss & Schmitt, 2018, for a recent review). Indeed, similar sex-asymmetric trade-offs between physical and socioeconomic characteristics have been reported in actual partner choices. For example, women, but not men, are more likely to tolerate unattractive physical characteristics in a wealthier partner (Chiappori, Oreffice, & Quintana-Domeque, 2012; but see Oreffice & Quintana-Domeque, 2010). Since sex differences in these aspects of mate preferences have been reported for many different cultures (Buss et al., 1990; Buss & Schmitt, 2018), some researchers have suggested they most likely reflect evolved preferences for the types of mates that will maximize an individual’s reproductive fitness (Buss et al., 1990; Buss & Schmitt, 2018; Lippa, 2007).

Social role theory presents an alternative to this evolved preferences explanation for sex differences in preferences for good earning capacity and physical attractiveness (Eagly & Wood, 1999). Under social role theory, these sex differences are hypothesized to reflect the effects of the different social roles imposed on men and women (Eagly & Wood, 1999). Support for this account comes from reanalyses of early work on sex differences in mate preferences (Buss et al., 1990) that suggested sex differences in preferences for good earning capacity and domestic skills (housekeeping and cooking), but not physical attractiveness, were smaller in countries that scored higher on United Nations’ measures of gender equality (Eagly & Wood, 1999). Although these results were partially replicated by Zentner and Mitura (2012) and Kasser and Sharma (1999), Gangestad, Haselton, and Buss (2006) suggested Eagly and Wood’s (1999) findings for gender inequality were an artifact of “Galton’s problem” (i.e., autocorrelation across geographically close regions).

Given the controversy around the claim that sex differences in mate preferences covary with country-level differences in gender equality, we sought to replicate Eagly and Wood’s (1999) results in a new data set. By contrast with Eagly and Wood (1999), who used aggregated data to calculate sex difference scores at the country level, we used multilevel models to analyze the mate preferences for individual participants (see Lee, DeBruine, & Jones, 2018; Pollet, Tybur, Frankenhuis, & Rickard, 2014, for detailed discussion of why the latter approach is preferable because it takes into account variability in preferences within each country).

## Method

### Participants

A total of 5,399 participants completed one or more mate preference tasks. Of these, 927 participants were removed from the data set for either not reporting their age, reporting an age below 16 years, or reporting an age above 60 years. A further 1,212 participants were removed from the data set for not reporting to be exclusively heterosexual. This resulted in a sample of 910 men and 2,350 women (mean age = 23.90 years, *SD* = 7.82 years). Participants were not compensated for taking part in the study. Each participant reported what country they live in (number of countries = 36; Argentina, Australia, Austria, Belgium, Brazil, Canada, Chile, Croatia, Denmark, Finland, France, Germany, Greece, India, Indonesia, Iran, Ireland, Italy, Lithuania, Mexico, the Netherlands, New Zealand, Norway, Philippines, Poland, Portugal, Romania, Russia, South Africa, Spain, Sweden, Switzerland, The former Yugoslav Republic of Macedonia, Turkey, United Kingdom, and the United States).

### Mate Preference Tasks

Participants completed the trait-rating mate preference task and/or the trait-ranking mate preference task originally used by Buss et al. (1990) and reanalyzed in Eagly and Wood (1999). Five hundred thirteen participants completed only the trait-ranking mate preference task, 93 participants completed only the trait-rating mate preference task, with the remainder (*N* = 2,654) completing both the trait-rating mate preference and the trait-ranking mate preference task. For participants who completed both tasks, task order was fully randomized.

In the trait-rating mate preference task, participants were asked to rate the following attributes for how important they are when choosing a romantic partner using a 4-point scale (3 = *indispensable*; 2 = *important*, *but not indispensable*; 1 = *desirable*, *but not very important*; 0 = *irrelevant or unimportant*): good cook and housekeeper; pleasing disposition; sociability; similar educational background; refinement, neatness; good financial prospects; chastity (no previous experience in sexual intercourse); dependable character; emotional stability and maturity; desire for home and children; favorable social status or rating; good looks; similar religious background; ambition and industriousness; similar political background; mutual attraction—love; good health; education and intelligence. The order in which traits were presented for rating was fully randomized.

In the trait-ranking mate preference task, participants were asked to rank the following traits on their desirability in someone you might marry (1 = *most desirable trait*, 13 = *least desirable trait*): kind and understanding, religious, exciting personality, creative and artistic, good housekeeper, intelligent, good earning capacity, wants children, easygoing, good heredity, college graduate, physically attractive, and healthy. The initial order in which the traits were presented for ranking was fully randomized. Trait rankings were reverse scored so that higher scores for a given trait indicated stronger preferences.

Following Eagly and Wood (1999), we only analyzed preferences for good earning capacity, physical attractiveness, and domestic skills. For the trait-rating task, these traits were operationalized as ratings for “good financial prospects,” “physically attractive,” and “good cook and housekeeper,” respectively (following Eagly & Wood, 1999). For the trait-ranking task, these traits were operationalized as rankings for “good earning capacity,” “good looking,” and “good housekeeper,” respectively (also following Eagly & Wood, 1999). For the trait-rating task, 35 participants did not rate all three traits and were therefore removed from the data set prior to analyses.

### Gender Equality Measures

Participants took part in the study between 2011 and 2018. Gender equality for each country was estimated using the United Nations’ Gender Inequality Index (GII) and Gender Development Index (GDI). The GII measures gender inequalities in reproductive health (maternal mortality ratio and adolescent birth rates), empowerment (proportion of parliamentary seats occupied by females and proportion of adult females over 25 years with some secondary education), and economic status (labor market and force participation rate of female and male populations over 15 years). The GDI measures gender differences in development of health, knowledge, and living standards using the same component indicators as the Human Development Index. These measures were chosen because of their similarity to the Gender Empowerment Measure and gender-related development index used in Eagly and Wood (1999) and because Eagly and Wood’s social roles theory emphasizes the importance of the combined effects of gender inequality in economic, political, and decision-making roles. GII and GDI data were retrieved from http://hdr.undp.org/en/data. Lower scores on the GII and higher scores on the GDI indicate greater equality. For each participant, the GII and GDI scores used were matched to the year in which they participated. Because GII and GDI scores were not available for 2018, we used 2017 values for participants tested in 2018.

### Analysis

Analyses were carried out using R Version 3.4.0. Preferences for good earning capacity, physical attractiveness, and domestic skills were analyzed in separate mixed-effect models, as were preferences assessed using the trait-rating and trait-ranking tests. Analyses used linear mixed models with random effects of country and region, participant age and participant sex as predictors, and random slopes specified maximally (see Barr, Levy, Scheepers, & Tily, 2013). Participant age was standardized at the participant level and both GII and GDI were standardized at the country level prior to analyses. Participant sex was effect coded (female participants = −.5, male participants = .5). Following previous research on differences in behavior among countries (e.g., Lee et al., 2018), only responses from countries for which we had more than nine participants were analyzed. This left us with a sample of 2,986 participants from 36 countries for the ranking task and 2,524 participants from 30 countries for the rating data.

Following other recent work on differences in behavior among countries (Bulley & Pepper, 2017; Lee et al., 2018), we controlled for autocorrelation across geographically close regions (i.e., Galton’s problem) in follow-up analyses by including the United Nation’s geographic region classification in our models (in addition to country). All data (including trait ratings and rankings not analyzed here), analysis code, and the full specifications for each model are publicly available at https://osf.io/4sr5f/

## Results

**Figure 1.**
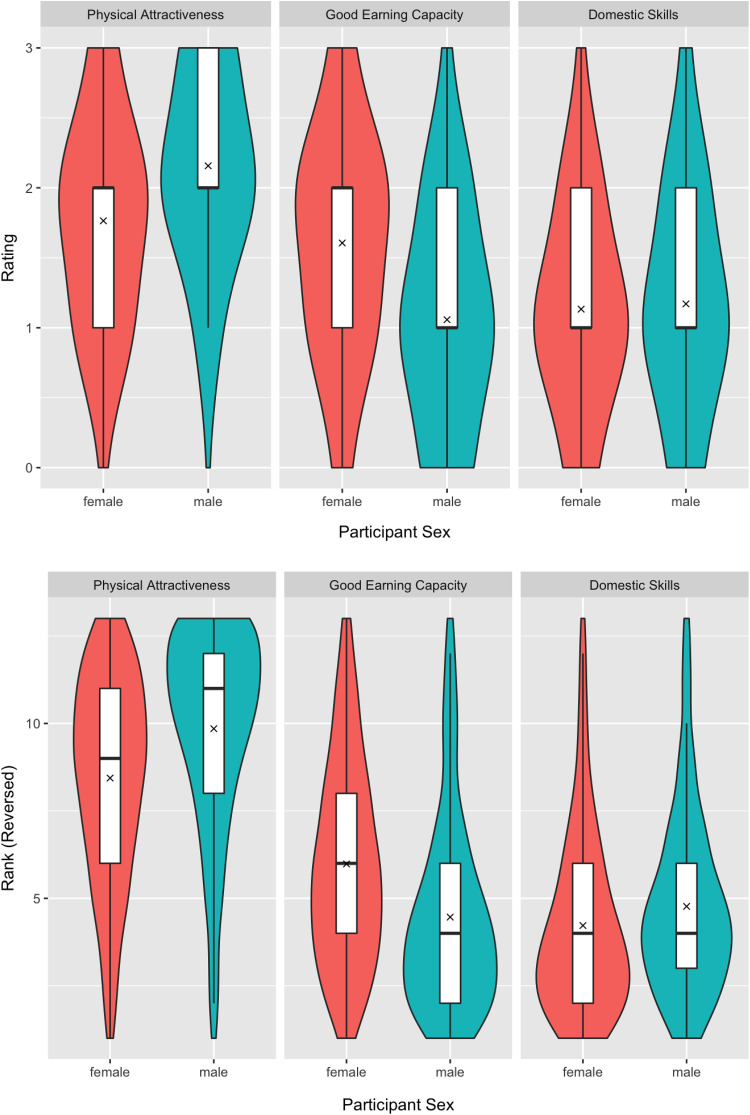
Violin plots showing men’s and women’s preferences for good earning capacity, physical attractiveness, and domestic skills in potential mates as assessed by responses on the trait-rating (top row) and trait-ranking (bottom row) tasks. Rankings have been reverse scored so that higher scores on both tasks indicate stronger preferences. The thick horizontal bar indicates the median and *x* indicates the mean.

We first tested for overall sex differences in preferences for good earning capacity, physical attractiveness, and domestic skills. Figure 1 summarizes men’s and women’s preferences for good earning capacity, physical attractiveness, and domestic skills in potential mates as assessed by responses on the trait-rating and trait-ranking tasks. Descriptive statistics for each country are given at https://osf.io/4sr5f/. Women showed stronger preferences for good earning capacity than men did for both ratings (estimate = −0.55, *t* = −11.16, *p* < .001) and rankings (estimate = −1.63, *t* = −5.96, *p* = .024). Men showed stronger preferences for physical attractiveness than women did for both ratings (estimate = 0.42, *t* = 9.25, *p* = .003) and rankings (estimate = 1.38, *t* = 7.90, *p* = .001). There were no significant effects of participant sex on the desirability of domestic skills in a potential mate for either ratings (estimate = 0.02, *t* = 0.52, *p* = .63) or rankings (estimate = 0.22, *t* = 1.40, *p* = .26). Full results for each of these models are given at https://osf.io/4sr5f/

**Figure 2.**
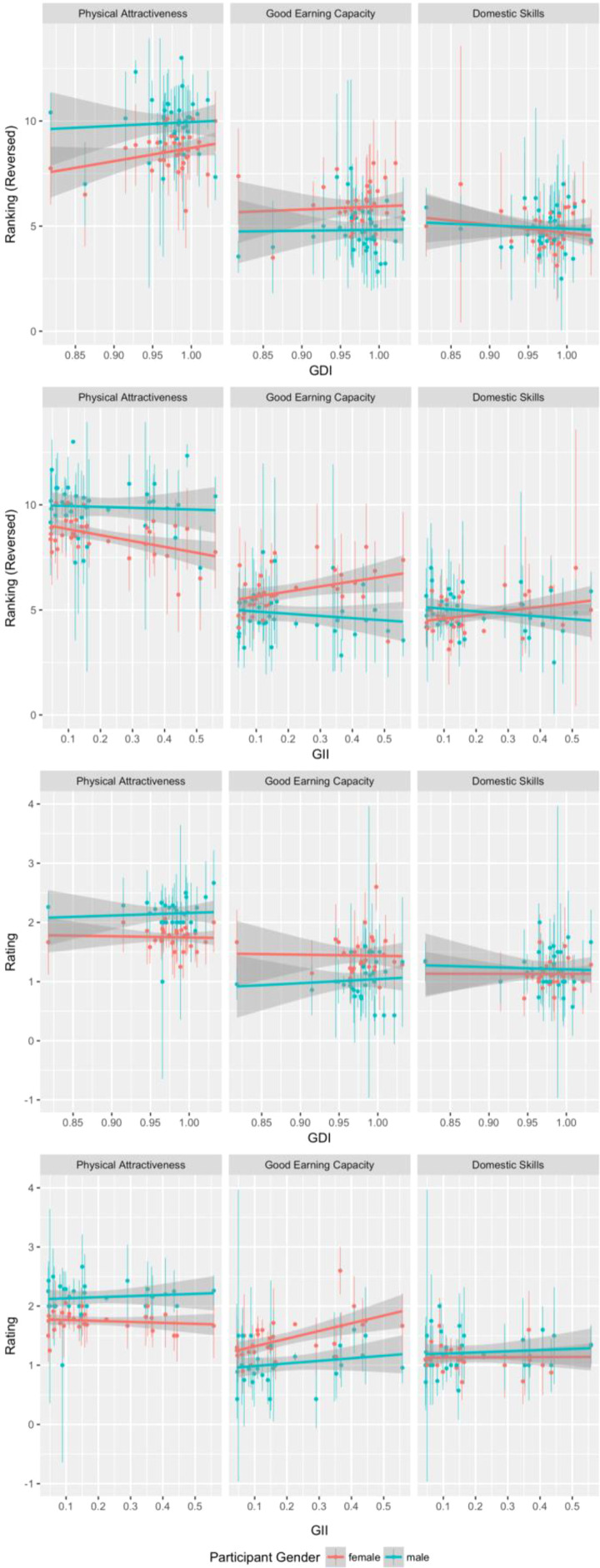
Interactions between participant sex and gender equality measures for each combination of trait and rating task. Dots show means and lines show SEM. Lower scores on the Gender Inequality Index and higher scores on the Gender Development Index indicate greater equality.

We repeated each of the models described above, this time including either GII or GDI as additional predictors, along with their two-way interactions with participant sex and participant age. Of the 12 models testing for possible effects of gender inequality, none showed a significant (i.e., *p* < .05) interaction between gender equality and participant sex (all absolute estimates <0.65, all absolute *t*s <2.10, all *p*s >.051). Full results for each of these models are given at https://osf.io/4sr5f/. Results of tests for the critical interactions between the effects of gender equality and participant sex are summarized in Table 1. Graphs showing each of these interactions are shown in Figure 2.

**Table 1.** Results of Tests for Interactions Between the Effects of Gender Equality and Participant Sex in Analyses Controlling for Galton’s Problem.

Trait	Gender Equality Measure	Task Type	Estimate	*t*	*p*
Physical attractiveness	GII	Rating	0.13	1.67	.10
Physical attractiveness	GII	Ranking	0.48	1.90	.06
Physical attractiveness	GDI	Rating	−0.09	−0.82	.41
Physical attractiveness	GDI	Ranking	−0.25	−0.60	.55
Good earning capacity	GII	Rating	−0.11	−1.33	.19
Good earning capacity	GII	Ranking	−0.64	−2.09	.06
Good earning capacity	GDI	Rating	0.04	0.31	.76
Good earning capacity	GDI	Ranking	0.24	0.56	.58
Domestic skills	GII	Rating	0.14	1.73	.09
Domestic skills	GII	Ranking	0.08	0.35	.73
Domestic skills	GDI	Rating	−0.05	−0.36	.73
Domestic skills	GDI	Ranking	−0.27	−0.68	.50

*Note*. GII = Gender Inequality Index; GDI = Gender Development Index.

Repeating these 12 tests for possible effects of gender equality on mate preferences, this time with world region removed from our analyses (i.e., not controlling for Galton’s problem), only altered results in one case (see https://osf.io/4sr5f/). This exception was the analysis of good earning capacity assessed using the trait-ranking method, for which there was a significant interaction between participant sex and GII (estimate = −0.65, *t* = −2.30, *p* = .027).

## Discussion

Our analyses of sex differences in the desirability of physical attractiveness and good earning capacity in potential mates replicate the sex differences reported in previous research (see Buss & Schmitt, 2018, for a recent review). Specifically, we found that women (on average) reported stronger preferences for good earning capacity than men did, while men (on average) reported stronger preferences for physical attractiveness than women did. These sex differences were strong, consistent across two methods for assessing mate preferences (responses on the trait-ranking and trait-rating tasks), and were present when controlling for variability in responses across countries and geographic regions. Collectively, these features of our analyses provide further evidence that robust sex differences in preferences for good earning capacity and physical attractiveness of potential mates are relatively stable across geographic regions. We found no evidence for sex differences in preferences for potential mates with domestic skills in our sample (see also Buss et al., 1990).

Although we found the expected sex differences in preferences for both physical attractiveness and good earning capacity, evidence that these sex differences were smaller in countries with greater gender equality was less convincing. We saw no evidence that the sex difference in preference for physical attractiveness was greater in countries with greater gender equality. One analysis suggested that the sex difference in preference for good earning capacity was smaller in countries with greater gender equality, but this was only observed for one combination of preference task and gender equality measure (responses on the trait-ranking method analyzed in relation to GII). This effect was also not significant when we controlled for Galton’s problem and would not be significant if α was corrected for multiple comparisons. Thus, we cannot discount the possibility that this relationship is a false positive. Collectively, these results provide little support for the social roles account of sex differences in mate preferences.

That we do not replicate previous results for gender inequality and mate preference sex differences is unlikely to be due to our study being underpowered relative to previous studies. We tested 36 countries, which is a similar sample size to the 37 countries tested in two of the previous studies (Eagly & Wood, 1999; Kasser & Sharma, 1999) and a considerably larger sample size than the 10 countries tested by Zentner and Mitura (2012). The null results in the current study also cannot be explained by the measures of gender inequality we employed. These are similar to those used in previous work on the topic that reported significant effects of gender inequality and, crucially, explicitly measure the combined effects of gender equality in economic, political, and decision-making roles that Eagly and Wood emphasized as being of critical importance for their observed effects. Indeed, while Eagly and Wood stated that using gender equality measures from different years than the preference data were collected was a limitation of their study, we matched our gender equality measures to the year in which preference data were collected (only substituting 2017 gender equality data for 2018 data because the 2018 data were not available).

An important limitation of the current study (and of work on this topic, generally) is that we assessed participants’ preferences for traits in potential mates rather than the traits their actual partners possessed. Although some research suggests some aspects of mate preferences predict actual partner choices relatively well (see DeBruine et al., 2006, for a review), other work suggests that for highly desirable traits, the ability to translate preferences into actual partner choices depends on one’s own market value (Wincenciak et al., 2015). Whether gender equality predicts sex differences in partner choices is an open (and important) question.

In summary, we replicated previous reports that women (on average) show stronger preferences for good earning capacity in potential mates than men do, while men (on average) show stronger preferences for physical attractiveness in potential mates than women do. However, we did not replicate Eagly and Wood’s (1999) finding that sex differences in preferences for physical attractiveness and domestic skills are smaller in countries with greater gender equality. We saw some evidence that the sex difference in preference for good earning capacity was smaller in countries with greater gender equality, but this effect was inconsistent across measures of mate preferences and gender equality, was not significant when controlling for Galton’s problem, and would not be significant when α was corrected for multiple comparisons. Together, these results present little compelling evidence for the social role theory of sex differences in mate preferences.
